# Overview of Immune Response During SARS-CoV-2 Infection: Lessons From the Past

**DOI:** 10.3389/fimmu.2020.01949

**Published:** 2020-08-07

**Authors:** Vibhuti Kumar Shah, Priyanka Firmal, Aftab Alam, Dipyaman Ganguly, Samit Chattopadhyay

**Affiliations:** ^1^Department of Biological Sciences, BITS Pilani, K. K. Birla Goa Campus, Goa, India; ^2^National Centre for Cell Science, S. P. Pune University Campus, Pune, India; ^3^Indian Institute of Chemical Biology, Kolkata, India

**Keywords:** coronavirus, immune response, COVID-19, T cells, MHC presentation, HLA, memory T cell

## Abstract

After the 1918 flu pandemic, the world is again facing a similar situation. However, the advancement in medical science has made it possible to identify that the novel infectious agent is from the coronavirus family. Rapid genome sequencing by various groups helped in identifying the structure and function of the virus, its immunogenicity in diverse populations, and potential preventive measures. Coronavirus attacks the respiratory system, causing pneumonia and lymphopenia in infected individuals. Viral components like spike and nucleocapsid proteins trigger an immune response in the host to eliminate the virus. These viral antigens can be either recognized by the B cells or presented by MHC complexes to the T cells, resulting in antibody production, increased cytokine secretion, and cytolytic activity in the acute phase of infection. Genetic polymorphism in MHC enables it to present some of the T cell epitopes very well over the other MHC alleles. The association of MHC alleles and its downregulated expression has been correlated with disease severity against influenza and coronaviruses. Studies have reported that infected individuals can, after recovery, induce strong protective responses by generating a memory T-cell pool against SARS-CoV and MERS-CoV. These memory T cells were not persistent in the long term and, upon reactivation, caused local damage due to cross-reactivity. So far, the reports suggest that SARS-CoV-2, which is highly contagious, shows related symptoms in three different stages and develops an exhaustive T-cell pool at higher loads of viral infection. As there are no specific treatments available for this novel coronavirus, numerous small molecular drugs that are being used for the treatment of diseases like SARS, MERS, HIV, ebola, malaria, and tuberculosis are being given to COVID-19 patients, and clinical trials for many such drugs have already begun. A classical immunotherapy of convalescent plasma transfusion from recovered patients has also been initiated for the neutralization of viremia in terminally ill COVID-19 patients. Due to the limitations of plasma transfusion, researchers are now focusing on developing neutralizing antibodies against virus particles along with immuno-modulation of cytokines like IL-6, Type I interferons (IFNs), and TNF-α that could help in combating the infection. This review highlights the similarities of the coronaviruses that caused SARS and MERS to the novel SARS-CoV-2 in relation to their pathogenicity and immunogenicity and also focuses on various treatment strategies that could be employed for curing COVID-19.

## Introduction

The whole world is currently confronting a crisis situation that first appeared in late December 2019 as merely a few cases of pneumonia in Wuhan, China. The patients were exhibiting common symptoms like fever, dry cough, sore throat, breathlessness, and fatigue. Sample swabs from the oral cavity and anal region were collected along with the blood and Bronchoalveolar Lavage Fluid (BALF) from all seven of the patients, irrespective of their age and gender, which were then sent to the Wuhan Institute of Virology for further examination. As the outbreak initiated at the seafood market with the onset of winter, similar to that of the previous Severe Acute Respiratory Syndrome (SARS) infection, the scientists first screened the samples using pan-CoV qPCR primers. Surprisingly, five samples were reported positive for coronavirus. Thorough investigation employing next-generation sequencing and phylogenetic analysis led to the identification of the causative agent of this respiratory disease, a novel coronavirus (2019-nCoV) (1). As more cases started to appear around the world, on February 11, 2020, the World Health Organization assigned a name, **CO**rona **VI**rus **D**isease 20**19** or COVID-19, to the disease and declared it a pandemic on March 11, 2020. The virus was renamed from 2019-nCoV to SARS-CoV-2 by the International Committee on Taxonomy of Viruses on the basis of its genetic similarity to a previously known coronavirus, Severe Acute Respiratory Syndrome Coronavirus (SARS-CoV) (2). Transmission of SARS-CoV-2 occurs when a healthy individual inhales or comes into contact with respiratory droplets from an infected person. The average incubation period before patients exhibit disease symptoms ranges from 2 to 14 days (3). Before the spread of COVID-19, SARS emerged as an epidemic in 2003, followed by Middle East respiratory syndrome (MERS) in 2012, both caused by a novel coronavirus of zoonotic origin and assigned to the genus Betacoronavirus (4). The worldwide outbreak of SARS-CoV-2 has put life on hold, having a major impact on the world's economy, and has claimed ~436,167 lives globally as of June 15, 2020 (5, 6). Unlike previous episodes of coronavirus spread, where it took months to identify the cause of infection and perform genome sequencing (7), advancement in science and technology made it possible to identify the causative organism swiftly. Within a few weeks of the outbreak, different laboratories across the world had sequenced the whole viral genome and had also provided structural and functional insights into the essential proteins required by the virus for its survival. These immediate scientific inputs helped with developing diagnostic kits and defining treatment strategies for effective prognosis and prevention (8–10). In this review, we are emphasizing the immunological aspect of SARS-CoV-2 pathogenesis by taking into consideration the previous experimental and clinical knowledge obtained from the coronaviruses that were responsible for causing SARS and MERS. This approach will assist in utilizing immunotherapies, repurposing the previously approved antiviral drugs, and developing therapeutic vaccines specific to novel coronavirus more effectively.

## Classification and Comparison of SARS-CoV-2

Initial genome sequencing and phylogenetic analysis of novel coronavirus SARS-CoV-2 has shown that it is genetically similar to previously known coronavirus SARS-CoV and hence is placed under the family *Coronaviridae*. Coronavirus contains positive-sense single-stranded RNA (+ve ssRNA) as its genetic material, which can be about 30 kb in length and is mostly protected by an outer fatty layer of an envelope that also helps the virus to evade host immune response and assists its entry inside the host cell (11, 12). The subfamily *Coronavirinae* is further subdivided into four genera, namely alpha-, beta-, gamma-, and delta- coronavirus (α-CoV, β-CoV, γ-CoV, and δ-CoV). Viruses having the potential to infect humans are placed under the genus α-CoV and β-CoV (SARS-CoV & MERS-CoV), whereas viruses of γ-CoV and δ-CoV genera are mostly known to infect avians and pigs (13). The novel coronavirus, SARS-CoV-2 falls under the genus β-CoV, as it shares 88% sequence identity with SARS-CoV-like coronaviruses (derived from bat) but is only 79% identical to SARS-CoV and 50% identical to MERS-CoV (3). Thus, it can be deduced by its genome identity that the immediate host of this virus could be a bat, which then transmitted it to some unknown intermediate host that acted as a source for the transmission of the virus to humans.

**Figure 1:**
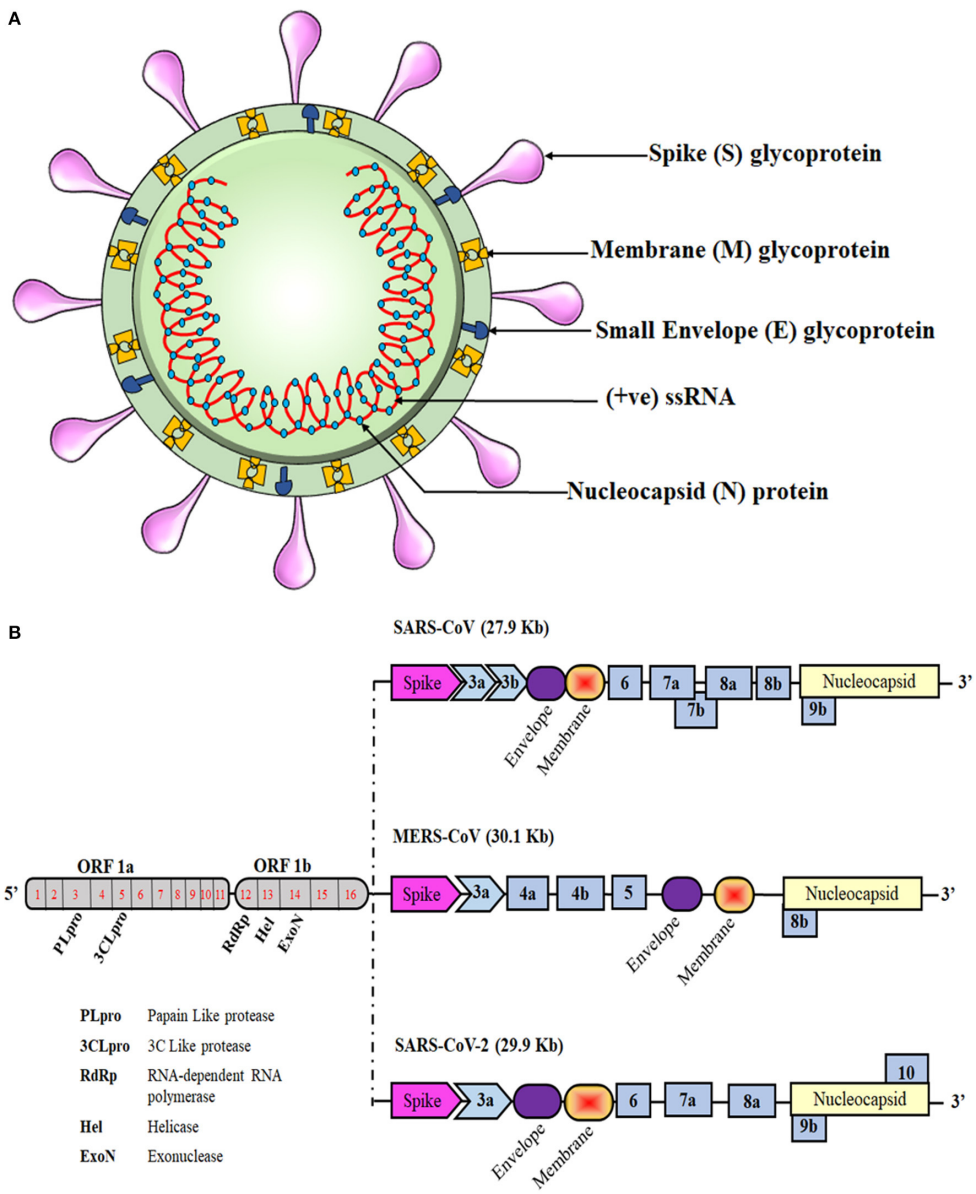
Schematic representation of the coronavirus structure and genomic comparison of coronaviruses. **(A)** Representation of coronavirus showing different components of the particle, which is 100–160 nm in diameter. The single-stranded RNA (ssRNA) genome, covered with the envelope and membrane proteins, gains access into the host cell and hijacks the replication machinery. **(B)** The ssRNA of SARS-CoV-2 is about 30 kb and has similarities with the genomes of SARS-CoV and MERS-CoV. Translation of this ssRNA results in the formation of two polyproteins, namely pp1a and pp1ab, that are further sliced to generate numerous non-structural proteins (NSPs). The remaining ORFs encode for various structural and accessory proteins that help in assembly of the viral particle and evading immune response.

Like those of SARS-CoV and MERS-CoV, the SARS-CoV-2 genome comprises of 12 open reading frames (ORFs) in number. At the 5′ end of the viral genome, overlapping ORFs 1a and 1b are present that encode the RNA polymerase and other non-structural proteins of the virus and occupy approximately two-thirds of the genome. Genes encoding structural proteins such as spike (S), membrane (M), envelope (E), and nucleocapsid (N), are present in the remaining one-third of its genome spanning from the 5′ to the 3′ terminal, along with several genes encoding non-structural proteins (NSPs) and accessory proteins scattered in between, as shown in Figure 1. Despite being in the same serogroup, there is a slight difference in the nucleotide number, sequence, gene order, and expression method among previously known coronaviruses and the novel SARS-CoV-2 (1, 14, 15). Recent reports highlight that a few amino acid substitutions have occurred in the novel coronavirus genes encoding the S protein, NSP2, NSP3, and receptor-binding domain (RBD). These mutations in the NSP2 & NSP3 are also believed to impart the enhanced infection abilities of the novel coronavirus (16, 17). RNA viruses are prone to acquiring genetic mutations that eventually help them to escape the host immune system and develop drug resistance. Researchers have also found minor mutations in SARS-CoV-2 genotype in different COVID-19 patients (18). One such hotspot of mutation in the SARS-CoV-2 genome is the RNA-dependent RNA polymerase gene. On analyzing 220 sequences across the globe, eight repetitive novel point mutations were observed. Viral genetic sequences accessed from Europe exhibited five mutation hotspots, whereas the remaining three point mutations were solely present in the sequences from North America. These unique mutations suggest that the viral strains are continuously evolving across the globe and that the strains from Europe, North America, and Asia might have co-existed the whole time (19). Another similar report analyzed 7,666 global viral genomic sequences and found 198 unique mutation sites on SARS-CoV-2 genome that encodes NSPs and S protein, suggesting that the virus is trying to adapt to its new host (20). As numerous drugs are currently being designed to target the proteins that are essential for the survival of the virus, rapid genetic mutation occurring in these proteins might not prove to be a potential candidate for drug design. Therefore, the invariable region of the virus could be a better target to avoid drug failures.

Interestingly, SARS-CoV-2, similar to SARS-CoV, exploits the angiotensin-converting enzyme 2 (ACE2) receptor to gain access inside human cells, whereas MERS-CoV binds specifically to Dipeptidyl Peptidase 4 (DPP4) receptor (21, 22). Binding of the virus particle to the specific receptor on the host cell plays a key role in governing its pathogenicity. Functional evaluation was carried out to reveal the potential receptors for different Betacoronaviruses (β-CoV) including SARS-CoV-2, and it was found out that the entry of the virus particle was enhanced in human cells expressing ACE2 receptor instead of DPP4 or Aminopeptidase N (APN) in the case of the novel coronavirus (23). Recent structural insights provided by Cryo-EM studies of S protein in prefusion conformation highlighted that the binding efficiency of ACE2 and S protein of SARS-CoV-2 is 10–20 times greater than for the previously known SARS-CoV (24, 25). The latest reports suggest that the trimeric S protein of SARS-CoV-2 is sliced by transmembrane protease serine 2 (TMPRSS2), similar to SARS-CoV (26, 27). Hence, profound knowledge of the potential receptors to which the virus particle can bind and its associated proteases will help us in designing specific antiviral drugs and neutralizing antibodies and will lead us to foresee whether particular coronaviruses of zoonotic origin could be able to adapt and infect humans.

## Coronavirus Replication

All coronaviruses initiate entry inside the target cell by engaging the host receptor with the S glycoprotein present on their surface so as to gain entry inside the target cell. The region of S protein containing the RBD is present on the S1 subunit. In a few coronaviruses, RBD is present at the N-terminus region of S1, whereas in SARS-CoV, it is situated at the C-terminus region (28, 29). The fusogenic activity of virus-cell membrane is governed by two tandem domains, heptad repeats (HR1,2) that are present on the S2 region of S protein (30, 31). Initially, it was believed that SARS-CoV enters the target cell merely by virtue of cell membrane integration of virus particle and host cell membrane (32). Later, it was discovered that an essential proteolytic cleavage event takes place in the S protein at the S2 position of SARS-CoV that results in membrane fusion and facilitates virus entry inside the cell (33).

Once the coronavirus is inside the host cell via membrane fusion, it releases its +ve ssRNA genome into the cytoplasmic compartment, where the translation of ORF-1a and ORF-1b begins resulting in the formation of two large polyproteins (pp1a and pp1ab). Three functional proteases then cleave the polyproteins into 16 non-structural proteins (NSP1-16), which eventually create the viral RNA polymerase and other accessory proteins for virus assembly (34–36). An uninterrupted replication-transcription event results in the formation of various nested sets of subgenomic (sg) mRNAs that eventually translate into numerous structural and accessory proteins (37). The E glycoproteins after synthesis are incorporated into the rough endoplasmic reticulum or Golgi membrane. The +ve ssRNA combines with capsid protein to form the nucleocapsid, followed by budding of assembled virus particles in the ER-Golgi Intermediate Compartment (ERGIC) (38). Lastly, the virus particle-loaded vesicles are fused with the cell membrane for effective shedding of the virus (4). These new virions are now accessible to infect the neighboring healthy cells and are also released into the surrounding environment via respiratory droplets that are highly contagious and hence potentially spread the disease to healthy individuals.

## Pathogenesis of COVID-19

The path followed by SARS-CoV-2 to reach the lungs is via the naso-oral cavity. Once the virus is inhaled, it enters the epithelial cells of the nasal cavity by engagement of ACE2 receptor with the viral RBD and initiates its replication (27, 39, 40). This initial asymptomatic phase lasts for about 1–2 days, during which the virus multiplies in the upper respiratory tract, where no major hindrance is caused by the innate immune cells. Within 2–14 days of initial encounter, the common symptoms of COVID-19 start to appear, which are similar to those of SARS and MERS, i.e., fever, dry cough, pharyngitis, shortness of breath, joint pain, and tiredness. Numerous problems arise during this phase of the disease, including nosocomial and fomite transmission of infection, which enhances the chances of community spread (41). Soon, the virus begins to move toward the lower respiratory tract via airways, and this triggers a strong innate immune response. Patients at this stage start exhibiting enhanced pro-inflammatory response that leads to viral sepsis accompanied by other complications, including pulmonary edema, Acute Respiratory Distress Syndrome (ARDS), different organ failures, and death in the worst scenarios (42). The infected individuals rarely show the intestinal symptoms like diarrhea that were evident in other coronavirus infections. Patients are recommended to be quarantined to prevent community spread of this pandemic virus (43). The severity of COVID-19 has been found to be greater in aged individuals and in people with a health history, such as those immune-compromised by HIV infection or by chemotherapy for cancer. Diabetic and asthma patients, along with individuals with hypertension, obesity, or heart, kidney, or liver disorders, are also at higher risk if they acquire the disease (44). Autopsy reports of individuals who died due to SARS show multi-organ dysfunction, with the highest viral titers in the lungs and immune cells in circulation, thus damaging the pulmonary and immune system (45, 46). As opposed to adults, only a very small population of children has been infected with SARS-CoV-2. In one study, the symptoms displayed by children above 15 years were found to be milder as compared to those of younger children, who showed severe symptoms but with rare deaths and better prognosis (47). The study speculated two major possibilities related to COVID-19 severity in children among different age groups. One of these rests on the finding that ACE2 activity is higher in children aged 4–13 years; after this age, it starts to decline until adolescence. This could be one of the reasons why lung fibrosis is observed mainly in younger children. Secondly, differential CD4^+^ and CD8^+^ T cell populations have been seen in children as compared to adults (48, 49). A large number of clinical and epidemiological criteria were defined to assess probable pediatric cases of COVID-19 (50). A preliminary report from a cross-sectional study of children admitted to US and Canadian Pediatric Intensive Care Units (PICUs) during March 14-April 3, 2020, revealed that the 48 children were admitted in the USA whereas no COVID-19 cases were reported in Canadian PICUs. The study revealed that there are fewer COVID-19 cases in children as compared to adults and that there is a median PICU time of 5 days (51). A recent preprint from Paris reports that 11 children (age 3.7–16.6) were admitted experiencing symptoms similar to Kawasaki disease (KD) along with gastrointestinal issues and elevated inflammatory markers. Further investigation suggested that they were also SARS-CoV-2-positive, speculating that this could be the reason for KD shock syndrome (52). Similar cases have been observed in New York, where four otherwise healthy SARS-CoV-2-positive children started displaying symptoms similar to KD and toxic shock syndrome, thereby needing intensive care (53). Therefore, medical practitioners should be prepared to tackle such sudden post-infection complications to avoid the associated risks.

## Immune Response to SARS-CoV-2

**Figure 2:**
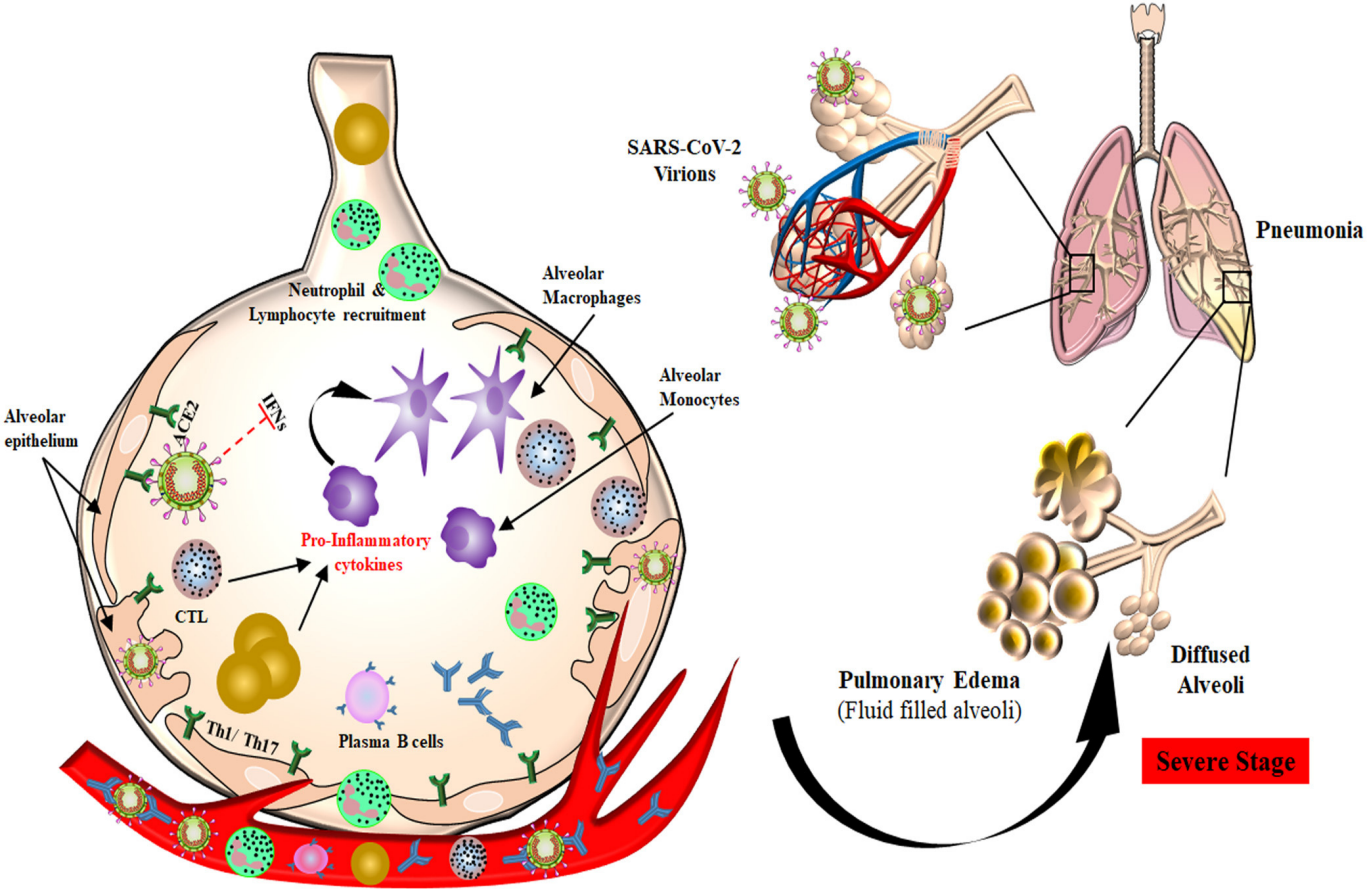
Plausible host immune responses during COVID-19 infection. The SARS-CoV-2 virus infects through the naso-oral route, followed by infection in cells expressing ACE2 receptor in the lung, such as type 2 alveolar cells. These viruses dampen anti-viral IFN responses by evading the innate immune cells as a consequence of unrestrained virus replication. The infiltration of monocytes/macrophages, neutrophils, and several other adaptive immune cells leads to increased pro-inflammatory cytokines. In the helper T cell subset, stimulation of Th1/Th17 cells with viral epitopes may lead to aggravated inflammatory responses. This inflammatory response results in “cytokine storms” that lead to immunopathologies like pulmonary edema and pneumonia. Cytotoxic T cells recruited to the site of infection try to kill virus-infected cells in the lungs. B cells/plasma cells also recognize viral proteins and are activated to produce antibodies specific to SARS-CoV-2, which may help in deactivating viruses and provide systemic immunity in different organs.

Once the virus gains access inside the target cell, the host immune system recognizes the whole virus or its surface epitopes, eliciting the innate or adaptive immune response (Figure 2). Pathogen recognition receptors (PRRs) present on immune cells, mainly Toll-like receptors 3, 7, and 8, are the first to identify the virus, which leads to enhanced interferon (IFN) production. The function of host innate immune cells is impaired during SARS-CoV and MERS-CoV infection by their non-structural proteins, which affects the overall cytokine production (54–56). Humoral response against SARS-CoV-2 has been found to be similar to that against other coronavirus infections, involving the characteristic IgG and IgM production. At the onset of SARS-CoV infection, B cells elicit an early response against the N protein, while antibodies against S protein could be detected after 4–8 days from the appearance of initial symptoms (57, 58). Although N protein is smaller than S protein, it is highly immunogenic, and the absence of glycosylation sites on it results in N-specific neutralizing antibody production at an early stage of acute infection (59). SARS-CoV-specific IgA, IgG, and IgM antibodies were detected after the onset of symptoms at different time points in infected patients. A persistent level of IgG was detected for a longer period, whereas IgM levels started to decline after 3 months (60, 61). In an observational case study of 16 SARS-CoV-2 patients, anti-S-RBD IgG was detected in all of the subjects, whereas anti-N IgG and anti-S-RBD IgM were detected in 15 patients and anti-N IgM in 14 patients (62). An ELISA-based time kinetics study to detect the COVID-19 specific humoral immune response showed that the patients produced IgM and IgG antibodies that did not cross-react with other human coronaviruses except SARS-CoV. IgM and IgA antibodies were detected 5 days after the onset of initial symptoms, whereas IgG was detected after 14 days (63). Another kinetic study of viral shedding and antibody detection was published in a preprint and reported the presence of higher IgG and IgM antibody titers in severe patients. They also observed that weak responders for IgG antibody had higher viral clearance than strong responders. This observation suggests that robust antibody response leads to disease severity while feeble response is associated with the elimination of virus (64). A case study on pediatric patients reports that 5 out of 6 children showed a protective humoral response, with neutralizing IgG and IgM antibodies targeting the N and S-RBD proteins of SARS-CoV-2 (65). These studies propose that IgM-based ELISA can be used for early diagnosis of patients along with qPCR techniques to improve the sensitivity and specificity of the technique.

In addition to neutralizing antibodies, which are defensive and useful, there are numerous non-neutralizing antibodies in the system that aid the infection of immune cells and APCs. Previously existing SARS-CoV antibodies may promote the viral infection in FcR-expressing cells (66). This ACE2-independent pathway of viral entry does not result in viral replication; rather, viral shedding by macrophages enhances inflammation and tissue injury by myeloid cell activation. This mechanism of viral entry through non-neutralizing antibody that results in aberrant activation of immune cells is called ADE (Antibody-Dependent Enhancement) (66, 67). ADE has been observed in a number of viral infections, including SARS and MERS. In the case of SARS, anti-S antibodies were observed to be involved in ADE to gain entry into FcR-expressing cells (68), while in MERS, a neutralizing Mab (Mersmab1) targeting RBD aided in MERS pseudo-virus entry via the DPP4 pathway (69). Although there is no clear evidence regarding ADE in SARS-CoV-2 infection, it is still necessary to consider all of the odds in the pursuit of developing vaccines and treatment regimens involving antibodies (70).

### Antigen Presentation

During viral infection, T cells also recognize the viral antigens presented by MHC class I [MHC; Human Leukocyte Antigen (HLA) in humans], which in turn promotes the cytokine release and cytotoxic activity of CD8^+^ T cells (71). But in some other cases, MHC class II is also found to present SARS-CoV peptides to CD4^+^ T cells. Due to the genetic polymorphism of HLA, some haplotypes, like HLA-B^*^07, HLA-B^*^46, HLA-DRB1^*^12 (72), and HLA-Cw^*^08 (73), are found to be more susceptible to coronavirus infection, whereas the HLA-DRB1^*^03, HLA-A^*^02, and HLA-Cw^*^15 haplotypes are protected from SARS-CoV infection (74). Similarly, HLA-DRB1^*^11 and HLA-DQB1^*^02 were found to be vulnerable to MERS-CoV infection (75). Additionally, MHC expression is also found to be reduced during the infection due to epigenetic modifications of downstream molecules (76, 77). So far, HLA association is not very well-identified for SARS-CoV-2 infection, and this could be crucial for the prevention and treatment of COVID-19. However, in a recent report, blood plasma from COVID-19 patients was able to block the expression of HLA-DR on CD14^+^ monocytes, which was restored effectively on inhibiting IL-6, suggesting that decreased HLA-DR expression in SARS-CoV-2 patients is due to the buildup of hyper-inflammatory conditions (78). Decrease in MHC expression is also evident in cancer cells, which is a mechanism by which they evade the immune response by epigenetically modifying calnexin promoter. But infection with influenza virus in these cancer cells results in enhanced MHC-I presentation due to the increased expression of chromatin remodeling proteins, which stabilizes p53 expression and hence augments the immune surveillance of cancer cells (79). Therefore, molecules that can upregulate chromatin regulators and increase the MHC-I expression could potentially be used for COVID-19. Most of the T-cell epitopes presented by MHC complex are derived from structural proteins such as the S and N proteins of the coronavirus in both humans and animal models, while the NSPs have regulatory effects on the signaling cascade (80, 81). T cells can be stimulated by 14 epitopes, most of which are observed to be located on ORF3 and the S protein in SARS patients (61). In a large cohort study during SARS-CoV infection, S protein was the only immuno-dominant epitope for CD8^+^ T-cell activation (61), whereas, in MERS, CD8^+^ response was against the S and N proteins along with some of the M/E epitopes (82). These T-cell epitopes have been tested in animal models by assessing the lung pathology and T-cell response upon infection in BALB/c and C57BL/6 mice (80, 83). The sequence of SARS-CoV-2 being more similar to SARS-CoV than to MERS-CoV, with no mutation in 19 epitopes, provides a prospective subunit vaccine for stimulating a strong T-cell response in COVID 19 patients (84). In a recent study, samples from 20 convalescing COVID-19 patients were analyzed to check the development of adaptive immune response during infection. The results highlighted that helper T cells were eliciting a robust immune response against S, M, and N protein. The effect of adaptive immune response on humoral immunity was also compared, where a strong CD4^+^ T-cell response against SARS-CoV-2 eventually resulted in an increase in anti-S-RBD-specific IgG and IgA antibody titer. Along with CD4^+^ T cells, immunogenic epitopes on S, M, and N proteins were also able to activate CD8^+^ T cells. However, such T-cell response was not specific to recovered patients only but was also present in 40–60% of the individuals who were not exposed to SARS-CoV-2. Further analysis showed that they had pre-existing cross-reactive CD4^+^ T cells, which might have been generated in response to some previous coronavirus infection. Hence, these T-cells could impart protective immunity in such individuals against SARS-CoV-2 to some extent (85). These epitopes could be a promising factor in developing immunotherapy by small molecules that can increase the presentation of viral epitopes.

### Cytokine Production

A rapid and coordinated immune response during viral infection leads to enhanced secretion of various cytokines, which acts as a defense mechanism against the virus. Numerous reports suggest that individuals affected with SARS-CoV or MERS-CoV have dysregulated cytokine production from both innate and adaptive immune cells. In the case of SARS, infected hematopoietic cell, monocyte-macrophages, and other immune cells trigger enhanced secretion of pro-inflammatory cytokines like TNF-α, IL-6, and IFN-α/-γ, with reduced anti-inflammatory cytokines (86–88). Similarly, MERS-CoV infection leads to delayed but increased production of IFN-α and pro-inflammatory cytokines like IL-6, IL-8, and IL-1β (89–91). Such elevated levels of cytokines were associated with Multi-Organ Dysfunctional Syndrome (MODS) and ARDS due to the accumulation of numerous immune cells like macrophages, neutrophils, and dendritic cells in the lungs causing alveolar damage and edema (56, 92, 93). Similarly, in COVID-19 patients, secretion of cytokines and chemokines, which attract the immune cells to the lungs, was increased, hence causing ARDS, which is fatal to critically ill individuals (94, 95). Signature cytokines in severely ill COVID-19 patients were consistent with those in SARS and MERS, i.e., enhanced expression of IL-6, TNF-α, macrophage inflammatory protein 1-α (MIP-1α), MCP3, GM-CSF, IL-2, and IP-10 along with elevated chemokines (IP-10, CCL2/MCP1, CXCL1, CXCL5) were also detected in SARS-CoV-2 infection (96–99). In children, the increased inflammatory markers include IL-6, IL-1, and C-reactive protein along with procalcitonin in serum (52). In a case study, a 14-year-old child with cytokine storm was treated with anakinra (IL-1 receptor antagonist) in order to stabilize the respiratory illness and other clinical symptoms (100). Transcriptomic analysis of PBMC and BALF showed that a number of immune regulators were upregulated, particularly CXCL10, with respect to BALF. This study also reported that several apoptotic genes and P53 signaling molecules were upregulated, suggesting a possible reason for lymphopenia in these patients (101). Therapeutic measures to control such cytokines involve neutralizing antibodies or small molecular drugs that can stop the signaling cascade for cytokine production.

### Immune Evasion

**Figure 3:**
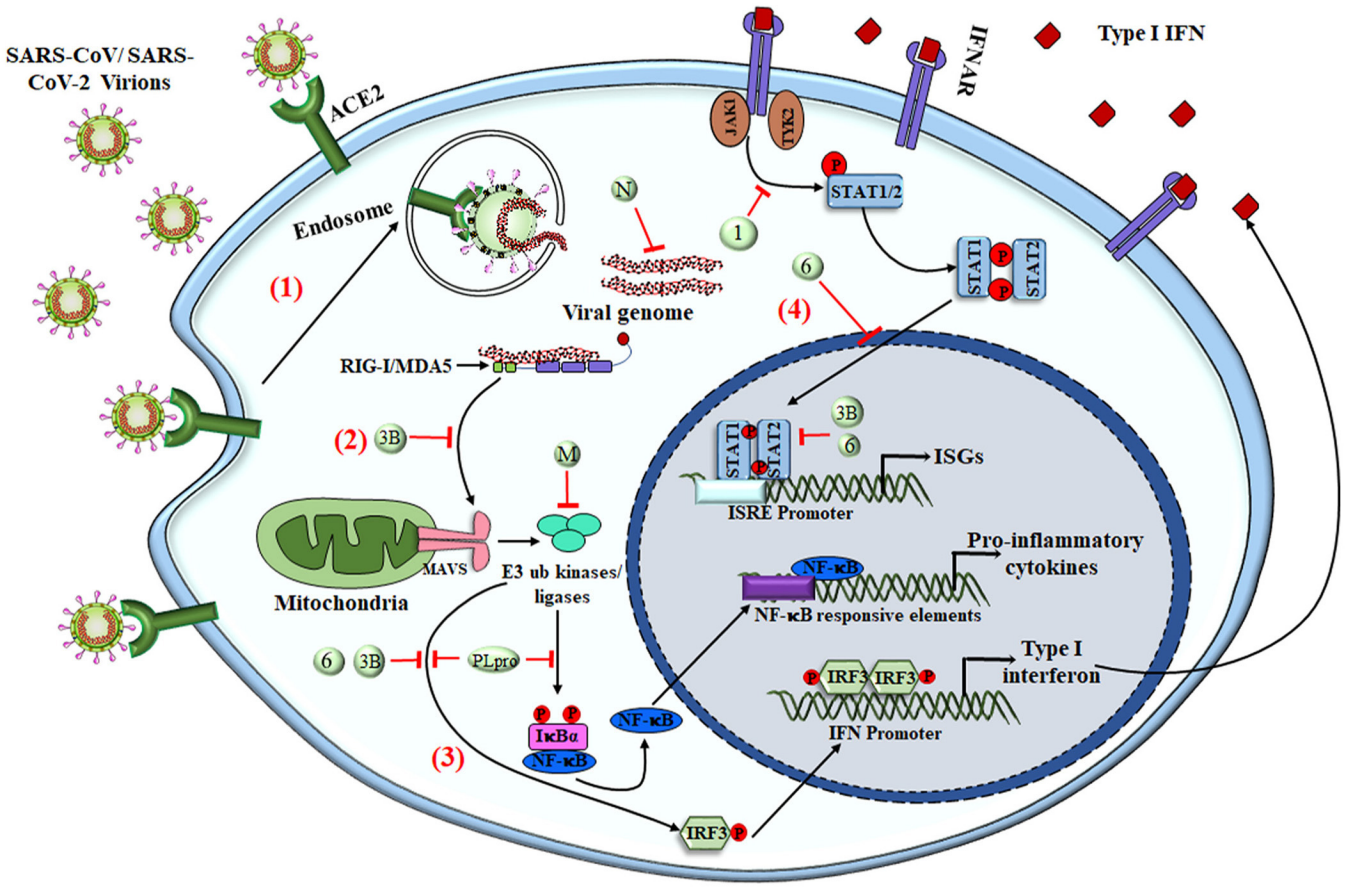
SARS-CoV-mediated evasion of host innate immune response. The viral antigens are recognized via different PRRs to elicit the innate immune response. (1) Upon interaction of the virus with surface PRRs or the specific receptor, the particles are endocytosed into the cytosol and are then recognized by cytosolic PRRs like RIG-I and MDA5. (2) The viral genome, along with different proteins, interacts with MAVS and initiates NF-κB activation via triggering a signaling cascade that involves numerous E3 ubiquitin kinases and ligases. (3) Upon translocation into the nucleus, activated NF-κB acts as a transcriptional activator for numerous pro-inflammatory cytokines with an NF-κB-response element. The IFN-regulatory factor 3 (IRF3), upon phosphorylation via ubiquitin kinases, homodimerizes and moves inside the nucleus to activate the transcription of Type I IFNs. (4) Type I IFNs have both autocrine and paracrine mechanisms to activate the JAK–STAT signaling pathway via IFNα/β receptor (IFNAR), followed by phosphorylation of STAT1 and STAT2 via cytoplasmic protein JAK1 and TYK2 kinases. STAT1 & STAT2 heterodimers translocate into the nucleus and are recruited for transcription of the IFN-stimulated gene having an IFN-stimulated response element (ISRE) present on their promoter. SARS-CoV and other coronaviruses have found many ways to inhibit the signaling cascade by utilizing either the structural proteins (M and N protein) or NSPs (NSP1, NSP3b, and NSP6 along with PLpro), shown as numbers and letters in the figure. Together, the production of pro-inflammatory cytokines and type I IFNs tries to create an antiviral immune microenvironment that controls viral synthesis and infection, but the viruses have deployed various strategies to shut down these signaling pathways to counteract the immune response. RIG-I, Retinoic acid-Inducible Gene I protein; MDA5, Melanoma Differentiation-Associated protein 5; MAVS, Mitochondrial antiviral-signaling protein; M, Membrane protein; N, Nucleocapsid; IFNAR, IFNα/β receptor; ISGs, IFN-stimulated genes; ISRE, IFN-stimulated response element.

The most potent antiviral machinery acquired by immune cells is the secretion of interferons that act as secondary messengers stimulating the neighboring cells. Most innate immune cells are efficient in producing IFNs that are involved in obstructing cell proliferation, apoptosis, and immunomodulation (54, 102). As an escape mechanism, SARS-CoV or MERS-CoV uses several ways to overcome the host immune response, one of which is by severe leukopenia and lymphopenia (103–105). After gaining entry to the cell, these viruses encode different proteins that interact with downstream signaling molecules of TLRs and the JAK-STAT pathway. MERS-CoV encoded matrix protein, accessory proteins from ORF 4a, 4b, and 5, which directly inhibits the IFN promoter and nuclear localization of IRF3 (106). PLpro, encoded by SARS-CoV and MERS-CoV, prevents the dissociation of NF-κB from IκBα, whereas nonstructural proteins of SARS-CoV, i.e., PLpro and ORF3b, inhibit IRF3 phosphorylation and hence its translocation to the nucleus (4, 107, 108). These viral accessory proteins also inhibit the JAK-STAT pathway, resulting in inhibition of genes by ISRE promoters (109–111) (Figure 3). A new investigation revealed that SARS-CoV-2 infection leads to an overall decrease in the transcription of antiviral genes because of the lower production of Type I and III interferons with sufficient ISG expression, along with elevated chemokine secretion. Results obtained from *in-vivo and ex-vivo* COVID-19 experiments were in tune with the *in-vitro* findings. Therefore, a decrease in the innate antiviral response, along with hyper-inflammation, could be one of the causes of COVID-19 severity (112). In addition to reduction in T cells, SARS-CoV-2 infection also enhances the exhaustion of effector T cells, decreasing the immune response against the virus (94, 113). Exhaustion and loss in function of effector T cells is the result of increased expression of inhibitory receptors like PD-1, TIM-3, and TIGIT on its surface as a result of cytokines like IL-6, IL-10, and TNF-α or by decreasing the regulatory T-cell population (114, 115).

### Memory T Cell

Following viral/antigen clearance, most of the effector T cell undergoes apoptosis in the contraction phase. Subsequently, a pool of memory T cells are generated that are programmed to fight against re-infection. CD4^+^ memory T cells, upon re-stimulation, trigger B cells and other immune cells by cytokine production, while cytotoxic memory T cells help in destroying the infected cells during subsequent infection (116, 117). Case studies in recovered SARS patients showed that both CD4^+^ and CD8^+^ memory T cells were efficient in eliciting immune response from 3 months to 6 years without the presence of any antigens (118). In a case study of 23 recovered SARS-CoV patients, the patients showed very low frequencies of memory B cells, while memory T cells elicited a response against the S protein in 60% of recovered individuals (119). Considering the memory T-cell subset, N-specific helper T cells had more of central memory markers (CD45RA^−^, CCR7^+^, CD62L^−^) while the CD8^+^ T cell population had the effector memory (CD45RA^+^, CCR7^−^, CD62L^−^) phenotype in a steady-state manner (120). The study suggests that an effective vaccine or T cell epitopes could be used to target a particular population for rapid viral clearance. In recent reports, COVID-19 subjects have shown reduced regulatory T cell populations and memory T cells, which may aggravate the inflammatory response leading to cytokine storm and hence enhance the tissue damage and organ failure (114). In a mouse model, the use of CD4^+^ memory T cells as a vaccine by the intranasal, but not the subcutaneous, route imparted a protective response against the human coronavirus. The infused CD4^+^ memory T cell, upon re-stimulation, produces IFN-γ and recruits CD8^+^ T cells for rapid clearance in response to SARS-S366 peptide (121). Recently, a human ACE-2-expressing mouse model has been developed by CRISPR/Cas9 technology that recapitulates the human symptoms upon infection with SARS-CoV-2 through the intra-nasal route. This tool will be beneficial for evaluating the efficacy of vaccines for COVID-19 and also to study its transmission and pathogenesis (122).

## Treatment Strategies for COVID-19

Just like SARS and MERS, there are no specific clinically approved drugs available for COVID-19 as of June 15, 2020 (123). Currently, the treatment regime focuses mainly on providing intensive care in order to alleviate the symptoms and discomfort associated with COVID-19. Conservative fluid therapy accompanied by broad-spectrum antibiotics are also given to the patients as a protective measure to avoid opportunistic bacterial infections. However, ventilator support for respiration is provided to the patient under extreme conditions (124). Numerous FDA-approved antiviral drugs, vaccines, and immunotherapies that are already being used to treat other diseases have also been considered as a possible approach for treating COVID-19 (Table 1). But this approach may reduce the availability of these drugs and vaccines for the intended diseases and for the patients with the greatest need. The molecular, structural, and functional relationships of SARS-CoV-2 with SARS-CoV might define the use of existing anti-viral drugs against COVID-19 (147, 148), considering the total time it takes to perform clinical trials and get FDA approval for the use of novel drugs and vaccines. The increasing knowledge of the genetic, immunological, and molecular mechanisms behind its enhanced pathogenicity might help in developing specific treatment approaches for COVID-19 in the future.

**Table 1:** List of drugs and vaccines for the treatment of COVID-19.

	**Targets**	**Description**	**References**
**MONOCLONAL ANTIBODY THERAPY**			
S230.15 mAbs m396 mAbs	RBD–ACE2 interaction	Tested in mice against SARS virus (strains Urbani, rGD03, or rSZ16).	(125)
MERS-4 MERS-27	RBD–DPP4 interaction	Blocks receptor–ligand interaction at the cell surface and prevents syncytia formation.	(126)
Tocilizumab	IL-6 receptor	Obstructs IL-6-mediated signal transduction.	(127)
Infliximab	TNF	Blocks soluble tumor necrosis factor and signal transduction, which helps maintain remission of COVID-19.	(128)
Adalimumab			
Lenzilumab	GM-CSF	Neutralization antibody for GM-CSF that is essential for chronic and acute inflammation in COVID-19.	(129)
Gimsilumab			(130)
**Interferons**	IFNß-1b	Enhances ISG expression via JAK/STAT signaling. Hinders virus multiplication and shedding.	(131)
	IFN- λ		(132)
**SMALL-MOLECULE ANTIVIRAL DRUGS**			
Aurine tricarboxylic acid	Viral RNA polymerase	Binds to viral polymerase, and tested against SARS virus in *in-vitro* culture.	(133)
Rupintrivir	Viral proteases	Protease inhibitor: inactivates 3CLpro and PLpro.	(134)
Benzopurpurin B	NSP15 endo-ribonuclease	Reduces viral infectivity of SARS virus in cell culture by inhibiting NSP15.	(135)
C-21	Angiotensin AT2 receptor	AT2 receptor agonist that may improve the viral damage to the lungs.	(134)
β-D-N4-hydroxycytidine (NHC)	Viral RNA polymerase	Inhibits replication of multiple coronaviruses. Can be used orally.	(136)
**REPURPOSED FDA-APPROVED DRUGS**			
Baricitinib	JAK kinase	Interferes with inflammatory signaling involving Janus kinase.	(137)
Lopinavir	Viral protease	Involved in immature, noninfectious HIV virus particle, and inhibits PLpro or 3CLpro in SARS-CoV-2.	(138)
Ritonavir	CYP3a (target unknown for coronavirus)	HIV protease inhibitor. No positive response in combination with lopinavir.	(139)
Favilavir	Viral RNA polymerase	Purine analog blocking viral RNA synthesis.	(140)
Remdesivir			(141)
Ribavirin		Guanosine nucleoside binds to nucleoside binding pocket of the enzyme.	(133, 140, 142)
Galidesivir		Adenosine analog, effective against Ebola, Zika, and other RNA viruses.	(143)
Chloroquine/hydroxychloroquine	Heme polymerase and ACE2	Increases endosomal pH and terminal glycosylation of ACE2, inhibiting SARS-CoV-2 entry.	(144, 145)
Nitazoxanide	Glutathione-S-transferase	Alters pH and inhibits viral maturation. Reported against TB, helminthic, and protozoan infection.	(140)
Umifenovir/arbidol	N/A	Interacts with aromatic residues of viral glycoproteins. Is being trialed for prophylactic action against COVID-19.	(146)

### Antiviral Agents

Considering the studies on the molecular mechanism of coronavirus infection (147), several antiviral drugs could be repurposed for the treatment of COVID-19. Remdesivir is a nucleotide analog that acts as an antiviral agent for a wide variety of viruses and has been tested widely against previous epidemics of coronavirus infections in both *in-vitro* and *in-vivo* models (138, 149–151). This adenosine analog gets incorporated into the newly synthesized viral RNA, which inhibits the addition of further nucleotides by viral RNA-dependent RNA polymerase and hence terminates the ongoing transcription. Administration of intravenous remdesivir was found to be effective in treating the first known patient of COVID-19 in the USA (152). A randomized double-blinded clinical trial on 1,059 adult hospitalized COVID-19 patients was sponsored by the National Institute of Allergy and Infectious Diseases, USA, to further test the potency of intravenously administered remdesivir. The preliminary outcomes of the trial reported that remdesivir treatment decreased the median recovery time in the treatment group (11 days) as compared to the placebo group (15 days). The mortality rate was also less in the treatment group (7.1%) in contrast to the placebo group (11.9%) (153). Numerous clinical studies, similar to this, are required so as to validate the proposed drugs for COVID-19. Favipiravir, ribavirin, and galidesivir are also potential nucleoside analogs that might be useful against novel coronavirus infection (154). The combinatorial therapy approach of using remdesivir along with chloroquine, a well-known anti-malarial drug, has also been tested *in vitro* so as to study its effectiveness against SARS-CoV-2 (141, 155). It has been reported that chloroquine immuno-modulates the host microenvironment and also interferes with the replication of the virus and its interaction with the receptor (156, 157). In a randomized clinical trial (NCT04308668) involving 821 asymptomatic individuals across the US and Canada who had come into close contact with potential COVID-19 patients, the individuals were given either hydroxychloroquine or placebo as a prophylactic measure. The results revealed that hydroxychloroquine treatment had the same effect as did the placebo group. The usage of hydroxychloroquine resulted in minor side effects (40.1%) as compared to the placebo treatment (6.8%). However, no cardiovascular disorder or treatment-related major complications were observed (158). Based on the putative function of hydroxychloroquine on the endosomal acidification, whereby it is presumed to hinder viral uncapping, it can be observed that it has a great potential for prophylaxis, not to prevent infection but to reduce effective viral load in patients and thus lead to milder disease. Numerous clinical trials to further explore the usage of hydroxychloroquine in different combinations are in the pipeline and will finally provide a better understanding of the efficacy of this drug for COVID-19. A few anti-HIV drugs, such as lopinavir/ritonavir in combination with interferon beta (IFN-β), have been tested *in vivo* for treating coronavirus infections (SARS-CoV, MERS-CoV) and have also been used in the case of COVID-19 (138, 139, 159). Various complementary therapies could also be employed as a preventive measure against viral infections. Many essential proteases, such as chymotrypsin (3C-like protease) and PLpro, which are required by coronavirus for completing the replication process, can also be targeted using drugs. Cinanserin, flavonoids, and some small molecules are known to inhibit 3CLpro, whereas diarylheptanoids are used to inhibit PLpro (160–162). In a recent study, 16 potential anti-HCoV drugs were identified through a systems biology-based approach, such as melatonin, mercaptopurine, sirolimus, dactiomycin, and toremifene, which are to be tested further for their potency (163).

### Plasma Therapy

In the absence of any dependable vaccine or drugs with tested efficacy and when the pandemic onslaught is ongoing, a worthy therapeutic approach is passive immunization using purified antibodies. The source of such antibodies could be the sera of convalescing individuals, mAbs, or genetically modified antibodies from an animal host, which can efficiently neutralize the virus. This is an age-old practice, with pioneering work having been done by the Nobel Laureate, Emil Behring, who applied this approach for diphtheria, and has been used whenever there are sudden outbreaks of viral diseases like SARS, MERS, H1N1, H5N1, Ebola, and many others (61, 164, 165). As opposed to active vaccination, plasma therapy is the only means to provide immediate immunity for viral clearance, as in the case of SARS-CoV-2. As in other epidemic diseases, convalescent sera are currently being employed for COVID-19 in a number of countries (166, 167). Although a randomized controlled trial is yet to be reported, limited studies in 10 patients have been documented with no remission of severe respiratory afflictions on receiving neutralizing antibodies from 39 convalesced donors with antibody titers of 1:160, along with drugs and oxygen support (168). A report from Hong Kong suggested that this therapy had poor outcome in SARS patients, with a number of limitations in their study (169). As with transfusion of any blood products, precautionary screening of infectious agent is warranted in plasma transfusion. Recently, the FDA in the USA has approved trials of convalescent plasma therapy in COVID-19 under specific guidelines; plasma donation is advised 3 weeks after a patient becomes virus-negative on PCR. The major challenge in this therapy is obtaining donors with similar blood antigens with a high antibody titer of SARS-CoV-2 (170). Another potential adverse effect of this approach is ADE of infection, which is common in so many other viruses. But, to date, the incidence of ADE has not been reported in the case of SARS-CoV-2. Another major point of contention is the selection of patients for this therapeutic approach. In most clinical trials, patients with severe diseases are being recruited, while the presumed mechanism of action of convalescent plasma, based on its content of virus-neutralizing antibodies, rather points to plausible favorable outcomes in earlier phases of the disease because in the later, more severe phases, the hyper-immune response, rather than the viral load, becomes the more critical pathology. Finally, there are no available data on the heterogeneity of response to convalescent plasma transfusion, which may further illustrate the importance of careful evidence-based patient selection, as heterogeneity of response may result from both virus and host-intrinsic factors which are, to date, not revealed.

### Vaccine Design Strategies

Researchers around the world are working hard to develop a potential vaccine candidate so as to stop the deadly pandemic caused by SARS-CoV-2. However, vaccine development is not an easy task, as a number of successful clinical trials are required before approval for patients. Different approaches are being utilized for designing a specific vaccine targeting either the structural proteins or viral replication process, which eventually results in the inhibition of viral growth and its further transmission. The common strategies involve the use of live attenuated vaccine (LAV), inactivated virus, subunit vaccines, monoclonal antibody vaccine, virus vectors, protein vaccines, and DNA/RNA-based vaccines (171–174). There are numerous subunit vaccines targeting all or a part of S protein that have already been tested for SARS and MERS in animal models (175) and could be potential candidates for testing against SARS-CoV-2. A recent pilot study with a purified inactivated SARS-CoV-2 virus vaccine displayed very promising outcomes in different animal models. The neutralizing antibodies generated after vaccination were able to effectively target 10 different strains of SARS-CoV-2 without developing any ADE of infection (176). Various randomized controlled trials (NCT04327206, NCT04328441) are also underway to evaluate the effectiveness of the BCG vaccine against SARS-CoV-2 for healthcare professionals. An adenovirus vector-based vaccine candidate, ChAdOx1 (presently AZD1222), developed by Oxford University (licensed to AstraZeneca) for use against SARS-CoV-2 has been reported to activate both the humoral and cell-mediated immune response when tested in rhesus monkey (177). The phase I clinical trial to confirm its potency is also in progress (NCT04324606). Another group has followed a similar approach by using a recombinant adenovirus type 5 (Ad5-nCoV) vector-based vaccine for COVID-19. The full report from the phase I clinical trial (NCT04313127) of Ad5-nCoV shows that it is very effective in generating both humoral and rapid T-cell response post immunization. The group is now ready for the next clinical trial phase to further strengthen the effectiveness of the Ad5-nCoV vaccine (178). It should be noted that there are potential risks associated with the usage of live attenuated viruses, for example, complications resulting in lung damage by infiltrating eosinophils, as seen in *in vivo* models (179, 180). However, eosinophil immunopathology due to SARS-CoV vaccine could be reduced by using TLR4 agonist as an adjuvant (181). Viral neutralizing antibodies specifically targeting various regions of S, i.e., S1-RBD, S1-NTD, or the S2 region, and blocking the interaction of virus with the receptor are well-known for SARS and MERS (182). These neutralizing antibodies could prove to be the best and potential candidate for cross-neutralization of SARS-CoV-2. Despite being structurally related, some of the SARS-CoV neutralizing monoclonal antibodies failed to interact with the S-protein of SARS-CoV-2, which could be attributable to the substantial differences in their RBD (183). A recent study reported the presence of high titres of neutralizing anti-S-RBD IgG antibodies, but no antibodies were detected against the N protein in recovered COVID-19 patients, suggesting that anti-S IgG persists longer than does anti-N IgG. Along with the humoral immune response, they also observed an S protein-specific T cell-population producing IFN-γ, which further contributes to conferring protective immunity against SARS-CoV-2 infection (184). Recently, a monoclonal antibody (47D11) has been identified from 51 SARS-Spike hybridomas that targets the conserved S-RBD region (residue 338–506) and therefore can very effectively neutralize SARS-CoV-2 along with SARS-CoV (185). On similar lines, a group has isolated a single-domain antibody from a phage display library targeting the S-RBD region of SARS-CoV-2. The fully humanized single-domain antibody was able to neutralize the virus by interacting with a cryptic epitope in S protein (186). These mAb and single-domain antibodies could be used to treat as well as to design quick diagnostic kits for COVID-19.

The new technology of the microneedle array (MNA) has been employed for delivering SARS-CoV-2 S1 subunit vaccine, which could be really helpful in the treatment of the emerging COVID-19 outbreak (187). The transfer of S1 subunit by MNA elicited a strong virus specific-antibody response in SARS-CoV-2 (187). A novel encapsulated mRNA vaccine candidate developed by ModernaTX, Inc. that encodes full length S protein of SARS-CoV-2, is also under clinical trial (NCT04283461). There is an urgent need to develop more such specific vaccines that could neutralize the novel coronavirus effectively (188).

### Immunomodulatory Therapies

The host innate immune system encounters upcoming infections, and this results in elevated production of various cytokines and type I interferons (IFNs). In the case of prolonged infection, hyperactivation of the immune system may also result in the development of a pro-inflammatory microenvironment, leading to adverse outcomes and even death. The induction of numerous lymphokines, such as IL-6, IL-1β, TNF-α, and CCL2, that are pro-inflammatory in nature has also been observed in the case of COVID-19 (189–191). A previous study in a MERS animal model showed that treatment with recombinant type-1 IFN (rIFN) decreased the viral RNA level in lungs with a decrease in IFN-stimulating gene expression. Early treatment with rIFN resulted in a dampening of cytokine and chemokine release that lowered the migration of neutrophils and other cells in lung (91). An allogenic mesenchymal stem cell-based (Remestemcel-L) therapy developed by Mesoblast, which has been previously used for inflammatory conditions and graft vs. host disease in children and adults, is now being assessed for COVID-19 (192–194). In this therapy, bone marrow-derived MSCs from the donor are grown *in vitro* and are then transfused to the recipient patients. Upon infusion, these cells exhibit anti-inflammatory activity by reducing pro-inflammatory cytokine production via the recruitment of anti-inflammatory cells in the affected tissue (195). Currently, a randomized placebo-controlled trial (NCT04371393) with 300 patients is ongoing for treating ARDS caused by COVID-19. Treatment with rIFN, inhibitors of the pro-inflammatory pathway, cytokine inhibitors such as tocilizumab, lenzilumab, and many others are still to be used in combination with other drugs for treating COVID-19. So far, there is not much evidence from clinical trials of such inhibitors with which to predict the outcome of these anti-cytokine therapies.

## Conclusion

Considering the current situation of more than 8 million people being infected, with ~436,167 deaths as of June 15, 2020, there is an urgent need to control the SARS-CoV-2 pandemic. The fatality rate of SARS-CoV-2 in lower than those of other coronaviruses that caused catastrophes in the past, but the higher infectivity rate makes it worse. Raising awareness of this contagious virus is one of the many ways by which its spread can be prevented. The governing authorities concerned in every country have approved guidelines and taken necessary action to quarantine infected people and break the chain of community spread. Antibodies, vaccines, and drugs developed for previously emerged coronaviruses could potentially be used for treating SARS-CoV-2. The combination of various neutralizing antibodies against S protein could enhance the effectiveness of viral clearance. Among various antivirals and other small molecules that are FDA approved, chloroquine/hydroxychloroquine has shown better positive outcome in COVID-19 patients. In clinical trials, some of the combinational antiviral drugs like lopinavir + ritonavir and blockers like angiotensin receptor blocker that were thought to be effective, have failed in curing the disease (139, 196). Cytokine storm being one of the symptoms of infected individuals, anti-cytokine therapy for TNF and IL-6 should be attempted to determine the efficacy of these antibodies in the treatment of SARS-CoV-2 infection. Clinical trial ChiCTR2000029765 with tocilizumab, a monoclonal humanized antibody against IL-6 receptor, has shown some efficacy, but this still needs to be tested in a larger cohort. With the increasing number of deaths, there is an immense need to accelerate the development of rapid and sensitive diagnostic kits and to commence clinical trials of the readily available and safe drugs to reduce the rising infections and COVID-19-related deaths so as to bring life back on track.

## Author Contributions

VS and PF contributed equally in writing the review. Conception of idea was done by SC, VS, and PF. Manuscript writing and editing was done by all the authors.

## Conflict of Interest

The authors declare that the research was conducted in the absence of any commercial or financial relationships that could be construed as a potential conflict of interest.
